# Norepinephrine Infusion in the Emergency Department in Septic Shock Patients: A Retrospective 2-Years Safety Report and Outcome Analysis

**DOI:** 10.3390/ijerph18020824

**Published:** 2021-01-19

**Authors:** Antonio Messina, Angelo Milani, Emanuela Morenghi, Elena Costantini, Stefania Brusa, Katerina Negri, Daniele Alberio, Ornella Leoncini, Silvia Paiardi, Antonio Voza, Maurizio Cecconi

**Affiliations:** 1 Humanitas Clinical and Research Center—IRCCS, Rozzano, 20089 Milano, Italy; angelo.milani@humanitas.it (A.M.); emanuela.morenghi@humanitas.it (E.M.); elena.costantini@humanitas.it (E.C.); stefania.brusa@humanitas.it (S.B.); katerinannegri@gmail.com (K.N.); daniele.alberio@humanitas.it (D.A.); ornella.leoncini@humanitas.it (O.L.); silvia.paiardi@humanitas.it (S.P.); antonio.voza@humanitas.it (A.V.); maurizio.cecconi@humanitas.it (M.C.); 2 Department of Biomedical Sciences, Humanitas University, Pieve Emanuele, 20090 Milano, Italy

**Keywords:** norepinephrine, emergency department, septic shock

## Abstract

Hemodynamic optimization during sepsis and septic shock is based on a prompt and large fluid resuscitation strategy associated with early administration of norepinephrine. In our hospital, norepinephrine is administered in the emergency department (ED), within a protocol-guided management context, to reduce norepinephrine infusion timing due to central line insertion. This choice, however, can be associated with side effects. Objectives: We conducted a retrospective analysis regarding the safety of norepinephrine in the ED. We also appraised the association between in-hospital mortality and predefined ED variables and patients’ admission severity scores. Design, settings, and participants: This was a retrospective analysis of electronic sheets of the ED of a tertiary hospital in the North of Italy. Outcomes measure and analysis: Electronic documentation was assessed to identify local and systemic side effects. We considered two subgroups of patients according to the in-hospital clinical paths: (1) those admitted in the intensive care unit (ICU); and (2) those who received a ceiling of care decision. We collected and considered variables related to septic shock treatment in the ED and analyzed their association with in-hospital mortality. Main Results: We considered a two-year period, including 108,033 ED accesses, and ultimately analyzed data from 127 patients. Side effects related to the use of this drug were reported in five (3.9%) patients. Thirty patients (23.6%) were transferred to the ICU from the ED, of whom six (20.0%) died. Twenty-eight patients (22.0%) received a ceiling of care indication, of whom 21 (75.0%) died. Of the 69 (54.3%) finally discharged to either medical or surgical wards, 21 (30.4%) died. ICU admission was the only variable significantly associated to in-hospital mortality in the multivariable analysis [OR (95% CI) = 4.48 (1.52–13.22); *p*-value = 0.007]. Conclusions: Norepinephrine peripheral infusion in the ED was associated with a low incidence of adverse events requiring discontinuation (3.9%). It could be considered safe within <12 h when a specific line management protocol and pump infusion protocol are adopted. None of the variables related to septic shock management affected in-hospital mortality, except for the patient’s ICU admission.

## 1. Introduction

Sepsis is a potentially life-threatening organ dysfunction caused by a host’s deregulated response to an infection [1]. Sepsis and septic shock (i.e., circulatory and cellular/metabolic dysfunction [2]) are primary healthcare problems affecting and killing millions of people all over the world [3], and thus they are a target of a worldwide effort to identify, document, prevent, and treat their occurrence [4,5]. Septic shock is nowadays considered a time-dependent syndrome, and the international bundles of resuscitation are mainly focused on source control (i.e., antibiotics administration and/or surgical treatment of the source) and shock reversal [1,2,6]. The latter is obtained via hemodynamic optimization based on a prompt and large fluid resuscitation associated with systemic vasopressors’ administration, specifically norepinephrine as a first choice [1,2,6]. The 2018 Surviving Sepsis Campaign Bundle recommends administering broad-spectrum antibiotics, rapidly administering 30 mL/kg crystalloid for hypotension or lactate ≥4 mmol/L, and applying norepinephrine if the patient is hypotensive during or after fluid resuscitation to maintain a mean arterial pressure ≥ 65 mm Hg within the first hour [7].

The central venous catheter (CVC) is commonly considered as the line of choice for vasopressor administration in the intensive care unit (ICU). However, the modality of norepinephrine administration in the emergency department (ED) is still a point of controversy [8,9]. In fact, on the one hand, the echography-guided process of CVC insertion is time-consuming and limited by the equipment available in the ED, potentially delaying norepinephrine administration. On the other hand, the use of peripheral venous lines may lead to extravasation and, consequently, to local tissue ischemia and injury [10,11].

At Humanitas Research Hospital (Rozzano, Milano, Italy), a tertiary hospital in the North of Italy, patients admitted for sepsis and septic shock in the ED may receive norepinephrine peripherally within a context of protocol-guided management, which includes an infusion pump connected to a dedicated vein at or above the antecubital fossa or to the external jugular vein, and continuous monitoring of vital parameters.

We conducted a retrospective two-year analysis regarding the use of norepinephrine in our ED, primarily to assess the occurrence of systemic or local side effects related to the drug infusion. Moreover, we appraised the association between in-hospital mortality and predefined ED variables (i.e., fluid infusion, norepinephrine dose, and timing of administration) and patients’ admission severity scores.

## 2. Materials and Methods

Data presented in this paper were retrospectively obtained from electronic health (wHospital^®^ Lutech Group, Milan—Italy) records of Humanitas Research Hospital (Rozzano, Milan, Italy), and the dataset of the outreach ICU team was recorded on a dedicated Excel^®^ (Excel 2011; Microsoft, Redmond, WA, USA) spreadsheet from 1 January 2018 to 31 December 2019. We excluded from the analysis all those patients who died early after ED admission (i.e., within 1 h) and those for whom the data regarding norepinephrine infusion were missing (i.e., dose and timing). The local ethics committee approved the use of these data.

### 2.1. Safety of Norepinephrine Infusion in the ED

Data on norepinephrine utilization include the minimal and maximum dose recorded, duration, and site of infusion. Norepinephrine preparation is standardized in the ICU and ED by diluting 4 mg in 50 mL of normal saline (80 mcg/mL) and infused by Alaris™ GH Plus syringe pump via a dedicated peripheral line. In our ED, the use of norepinephrine is restricted to selected high-dependency subunits (i.e., emergency room or observation bays) equipped with continuous non-invasive monitoring (including heart rate, peripheral oxygen saturation, continuous electrocardiography, and non-invasive blood pressure monitoring), with a patients/nurse ratio of 5:1.

Nursing and medical documentation were assessed to identify the evidence of either extravasation or unexpected/unjustified norepinephrine discontinuation. The patient chart was queried by two independent researchers (S.B. and An.Mi.) using the terms “extravasation,” “infiltration,” and “swelling” using a search function to identify extravasation events that were documented by any healthcare provider in the ED or after ICU admission. If any healthcare provider documentation identified extravasation, it was considered as a “confirmed extravasation.” If the patient’s line was discontinued without any specified reason and the chart reported the occurrence of “extravasation,” “infiltration,” “swelling,” or “problems related to line use” within the first 6 h after the discontinuation, then it was considered as a “possible extravasation.” In the case of disagreement between the two researchers regarding the occurrence of a “possible extravasation,” patients were independently reviewed, and whenever possible contacted, by a third senior author of the study (A.M.), for a definitive assessment.

We also considered any technical problem related to the pump use, including the incorrect infusion of a “bolus” of norepinephrine.

### 2.2. Sepsis and Hemodynamic Variables

We collected the Sepsis-related Organ Failure Assessment (SOFA) score and Charlson Comorbidity Index scores at admission. We assessed hemodynamic (i.e., mean arterial pressure (MAP)) and lactate values on admission and at 6, 12, and 24 h. We computed the total amount of fluids (including crystalloids, colloids, blood, or other hemoderivatives), the time from ED admission to norepinephrine administration, and the dose given. Finally, we collected the timing of empiric antibiotic administration and the source of infection.

### 2.3. Subgroups Definition

We stratified the patients according to the in-hospital clinical paths. Accordingly, we considered: (1) those admitted in ICU from the ED (NE_ICU_); and (2) those who received a ceiling of care decision (NE_CEI_) (i.e., do not escalate the level of care up to the Level 3 ICU beds). This last choice was discussed by the ED and ICU senior consultants and individualized considering patient wishes, the clinical frailty scores, the past medical history, and the patients’ condition at admission. Patients with a ceiling of care decision received all the medical and respiratory support required. If considered appropriate, end of life care pathways were started in the case of clinical deterioration.

### 2.4. Statistical Analysis

Normal distribution of continuous variables was evaluated employing the d’Agostino–Pearson test and, since some data failed the normality test, results were expressed as median (25th–75th interquartile range). Dichotomous or categorical variables were compared utilizing the chi-square test for comparison of proportions. Differences between the absolute value of MAP and lactate levels at the admission (baseline), 6, 12, and 24 h were ascertained using one-way analysis of variance (ANOVA) for repeated measurements, with the post hoc test of Dunnett for multiple comparisons: baseline was chosen as a control variable.

Association of discharge status (i.e., in-hospital mortality) and other variables were explored with a logistic regression analysis. All independent factors with a *p*-value less than 0.2 were then submitted to a multivariable logistic regression; we also corrected the multiple regression for important clinical variables, even if non-significant.

Statistical analyses were conducted using GraphPad PRISM V8 (GraphPad Software Inc., San Diego, CA, USA) and STATA15^®^. A *p*-value < 0.05 was considered statistically significant.

## 3. Results

In the two years considered, our ED had 108,033 accesses, of whom 14,328 (13.3%) were accounted for as admissions due to “infection” (any type). We identified 136 patients who received norepinephrine peripherally. We excluded nine patients from the analysis because one (0.7%) died within 1 h from ED admission and eight (5.8%) lacked data regarding norepinephrine dose administration. Ultimately, we analyzed data retrieved from 127 patients, in whom the source of the hemodynamic instability was due to pneumonia in 42 patients (33%), urinary tract infection in 41 patients (32%), abdominal infection in 22 (17%), catheter-related in 6 (5%), and other infections (i.e., surgical site and cutaneous ulcer) in 9 (7%). The source was considered unknown in seven patients (6%). General characteristics of the overall study population and the considered subgroups (NE_ICU_ and NE_CEI_) are reported in Table 1. In 11 patients (8.6%), a central line was inserted in the ED after starting norepinephrine peripherally, but none had reported a prior problem with peripheral drug infusion.

### 3.1. Safety of Norepinephrine Peripheral Use

According to the abovementioned indicators (see Methods Section 2.1), side effects related to the use of this drug were reported in five (3.9%) patients: a “confirmed extravasation” was reported in one (0.78%) patient. Additionally, in four (3.1%) patients, the line was discontinued because of “unspecified problems related to its use” (possible extravasation). No technical problems related to norepinephrine infusion were reported (i.e., unplanned bolus or wrong drug infusion targets).

### 3.2. Sepsis-Bundle Treatment

Overall, during the ED stay, patients received a median (IQR) of 4500 mL (3075–6250 mL) of fluids, corresponding to 72.5 mL/kg (44.0–101.7 mL/kg), and 11 patients (8.6%) received albumin in the context of shock reversal fluid therapy. No other types of colloids were administered. Twenty-five patients (19.6%) received antibiotic treatment within the first hour and 72 (56.6) within 3 h from ED admission. The median (IQR) time for antibiotic administration was 3.0 h (1.5–6.0). Patients in NE_ICU_ subgroup received earlier norepinephrine administration (*p*-value = 0.03) and a higher volume of fluids (7.3 mL/kg/h (2.9–19.5 mL/kg/h) vs. 3.6 mL/kg/h (2.8–9.2mL/kg/h); *p*-value = 0.01) during the ED stay, as compared to NE_CEI_ subgroup (Table 2).

**Figure 1:**
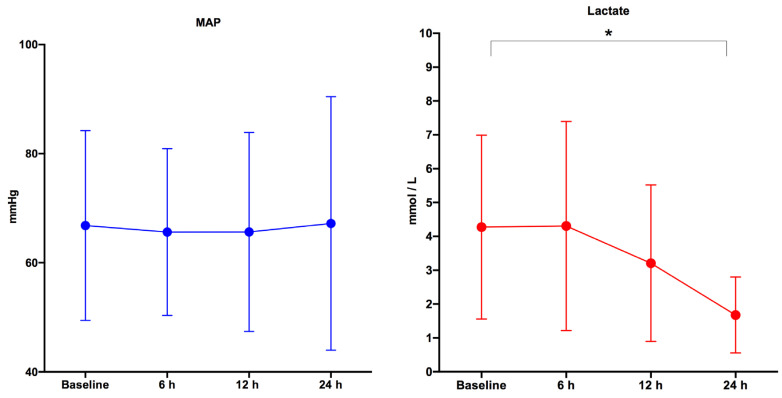
Mean arterial pressure (MAP) and lactate changes from baseline (ED admission) up to 24 h of the enrolled population. * = lactate level after 24 h was statistically lower than the baseline, *p*-value = 0.002

MAP did not change across the considered time-points (from baseline up to 24 h), while the lactate level after 24 h was statistically lower than the baseline (*p*-value = 0.002). (Figure 1).

### 3.3. In-Hospital Outcomes

Thirty patients (23.6%) were discharged to the ICU from the ED (NE_ICU_), and six (20.0%) died. Twenty-eight patients (22.0%) received a ceiling of care indication (NE_CEI_), and 21 (75.0%) died. Of the 69 (54.3%) finally discharged to either medical or surgical wards, 21 (30.4%) died. The mortality rate of these patients was not greater than the NE_ICU_ subgroup (*p*-value = 0.33). Overall, 48 patients who received norepinephrine in the ED died during the hospital stay (37.7%).

ICU admission was the only variable significantly associated to in-hospital mortality in the multivariable analysis [OR (95% CI) = 4.48 (1.52–13.22); *p*-value = 0.007] (Table 3).

## 4. Discussion

The main results of our retrospective two-year study regarding the use of norepinephrine in the ED for septic shock treatment can be summarized as follows: (1) peripheral infusion was associated with a low incidence of adverse events requiring discontinuation (3.9%) and could be considered safe within a period < 12 h and for low dosages, when a specific protocol of line management, patients’ monitoring, and pump infusion is adopted; (2) none of the variables related to septic shock management in the ED impacted on in-hospital mortality, except for ICU admission of the patient; and (3) even in the context of the ceiling of care clinical path, the ED administration of norepinephrine may be considered proportionate to revert an episode of septic shock.

Septic shock is a time-dependent life-threatening syndrome and a medical emergency, whose outcome is affected by early identification and appropriate immediate management [1,6]. The cardiovascular system is frequently affected by sepsis septic heart dysfunction is a key feature of sepsis-associated cardiovascular failure [12,13]. In fact, septic not-ischemic cardiomyopathy is a key feature of sepsis-associated cardiovascular failure, having different patterns of presentation (i.e., left ventricular dilatation with normal- or low-filling pressure and right or left ventricular systolic or diastolic dysfunction with a reduced response to volume infusion) [12,13]. Moreover, there is a strong association between impaired diastolic function and mortality in septic patients [14].

The urgent restoration of adequate perfusion pressure to the vital organs is a key part of resuscitation and is mainly obtained by a prompt and large infusion of fluids and norepinephrine as the first-choice agent [1,6,7]. Accordingly, the use of this vasopressor has been progressively shifted outside the ICU, in settings such as the ED, where few data are available regarding the safety, the duration of infusion, and the effects on the outcome of specific subgroups of patients.

### 4.1. Safety of Norepinephrine Use in the ED

Vasopressors are critical for managing patients in shock, and their prompt initiation has been associated with decreased mortality [15,16]. However, the literature concerning the use of vasopressors in the ED remains scarce. Historically, the reasons for limiting the use of these drugs outside the ICU are mainly based on avoiding the risk of potentially dangerous IV boluses associated with soft tissue damages. However, most of the clinical reports of extravasation and consequent skin necrosis after peripheral norepinephrine infusion are dated 50 years ago [11,17,18]. Modern IV pumps should minimize the risk of inappropriate bolus administration.

We reported a very low rate of side effects related to the use of this drug (i.e., overall 3.9% of patients, with only one “confirmed extravasation” (0.78%)), which are in line with the recent ED and ICU literature. Nuguyen et al. reported an overall rate of 4.5% of norepinephrine extravasation in the context of an ED standardized protocol of vasopressors infusion through peripheral venous lines in a retrospective cohort of 177 patients over three years, with no subsequent extremity injury [10]. In a different setting of 734 ICU patients, Cardenas-Garcia et al. reported an even lower extravasation rate (2%) without any tissue injury following treatment [19]. However, the duration of norepinephrine infusion in our patients [15.0 h (9.0–25.8)] was much longer than that reported by Nuguyen et al. [62 min (31–142)] [10]. Moreover, our patients were treated with a low dose of norepinephrine (Table 2), minimizing the risk of local damages.

For instance, difficulties in maintaining hemodynamic parameters during and after the changeover of vasoactive infusion pumps are common in the ICU [20]. However, considering the median ED dose, the timing of norepinephrine infusion (Table 2), and the standard preparation (see Section 2), pump syringes have probably rarely changed in our patients.

### 4.2. Use of Norepinephrine in the ED and Clinical Pathways

As shown in Table 2 the NE_ICU_ subgroup received an earlier norepinephrine administration [6.0 h (3.8–10.0 h) from ED admission vs. 11.5 h (4.2–13.7 h)] and a larger volume of fluids while in ED [7.3 mL/kg/h (2.9–19.5 mL/kg/h) vs. 3.6 mL/kg/h (2.8–9.2 mL/kg/h)], as compared to NE_CEI_ subgroup. Patients with a ceiling of care decision shared with the ED team accounted for 22% of the overall considered population. As expected, this subgroup had the highest mortality rate. However, 25% of NE_CEI_ patients did not die during the hospital stay, suggesting that the use of norepinephrine should not be considered inappropriate a priori for those patients admitted with septic shock but not suitable for Level 3 care.

The mortality of NE_ICU_ subgroup (20.0%) lays on the lowest range of the mortality reported in the literature (which ranges from about 20% to about 40% [1,21], being this heterogeneity mostly due to variability in defining and applying the diagnostic criteria, as well as differences in treatment and care across settings and countries), suggesting that ED norepinephrine administration did not delay proper treatment. However, ICU admission was the only variable associated with in-hospital mortality among all the others considered (Table 3), highlighting the efficacy of a prompt and aggressive ED approach to those patients admitted to Level 3 care. However, it should be underlined that ICU admission is not the cause of the increased mortality since this association is related to the underline severity of the septic shock and not the ICU admission itself.

### 4.3. Limitations

This study has some limitations that should be acknowledged. First, the retrospective analysis was primarily based on data obtained from the electronic ED clinical decision support software. For this reason, we could not report the exact timing of sepsis/septic shock assessment and diagnosis (and consequently the timing of the measurements adopted, such as the fluid and antibiotics treatment) for all patients. Some patients were admitted to the ED with a diagnosis of sepsis or septic shock by the triage nurse, while others deteriorated after a generic “infective” admission. Moreover, the retrospective design may lead to information bias related to the under-reporting of both the diagnosis of septic shock and potential complications related to the drug infusion. This limits our results’ external validity, which should be evaluated by using a prospective design with a predefined data collection.

Secondly, our results’ generalizability is strictly related to the local ED resources of each hospital. In fact, in our institution, norepinephrine administration acts as a warning bell, which notifies the ICU outreach team, formed by an anesthesia and intensive care specialist, which is available 24/7 to address urgent/emergent calls from the ED and wards. For instance, as suggested by the guidelines [1], all patients requiring vasopressors should have an arterial catheter placed as soon as possible if resources are available (weak recommendation). Strict clinical monitoring is needed when norepinephrine is used outside the ICU by the whole team involved in the patients’ management. This collaboration improves patient care and attention in decision-making processes such as initiation or discontinuation of norepinephrine or patient transfer to ICU according to bed availability.

Third, the ceiling of care decision plan was established after a multidisciplinary approach in the ED, which is, in our institution, mainly based on the frailty score assessment, because of its approved consensus among healthcare providers as described in the literature [22,23]. We could not extrapolate this score from the data available to differentiate the NE_CEI_ subgroup from the others.

## 5. Conclusions

In a protocol-based administration context, the peripheral infusion of norepinephrine in the ED could be considered safe within a period < 12 h and for a low-dose administration. Those patients admitted to the ICU are associated with the highest risk of in-hospital death and should be closely monitored while in the ED to optimize patient care.

## Figures and Tables

**Table 1:** Patients’ baseline characteristics at the ED admission.

Variables	Whole Population N = 127	NE_ICU_ N = 30	NE_CEI_ N = 28	NE_ICU_/NE_CEI_ Comparisons *p*-Value
General characteristics				
Age (years)	77 (69–85)	75 (64–78)	77 (70–83)	0.05
Males (*n*, %)	64 (50%)	18 (60%)	10 (36%)	0.07
BMI kg m^−2^	24 (21–26)	25 (22–26)	23 (20–26)	0.72
Charlson Comorbidity Index	2 (1–3)	1 (1–3)	2 (1–3)	0.26
SOFA score	4 (2–5)	5 (3–6)	4 (3–5)	0.54
Organ-related parameters				
HR (beats/min)	97 (80–120)	104 (84–121)	95 (79–110)	0.77
MAP (mmHg)	66 (53–78)	64 (54–74)	61 (52–73)	0.60
pH	7.46 (7.38–7.5)	7.46 (7.43–7.48)	7.41 (7.33–7.53)	0.66
Lactate (mg/dL)	3.5 (3.3–5.5)	3.0 (2.0–5.0)	4.0 (2.2–6.0)	0.57
Temperature (°C)	37.0 (36.0–38.0)	37.0 (36.0–38.0)	37.0 (36.0–38.2)	0.63
WBC (10^3^/mm^3^)	11.4 (4.4–18.9)	4.1 (1.7–14.1)	13.8 (6.9–19.3)	0.003
Respiratory rate (breaths/min)	18 (15–22)	18 (16–25)	18 (16–21)	0.58
Creatinine (mg/dL)	1.59 (1–2.62)	2.0 (1.4–2.7)	1.7 (1.0–3.1)	0.69

Values are presented as median (25th–75th interquartile range) or absolute (%). Group NE_CEI_, patients receiving a ceiling of care indication; Group NE_ICU_, patients admitted to ICU; BMI, body mass index; SOFA, sequential organ failure assessment; HR, heart rate; MAP, mean arterial pressure; WBC, white blood cells.

**Table 2:** ED sepsis bundle-related variables.

Variables	Whole Population N = 127	NE_ICU_ N = 30	NE_CEI_ N = 28	NE_ICU_/NE_CEI_ Comparison *p*-Value
Time to ED access to NE administration (h)	9.8 (4.0–15.5)	6.0 (3.8–10.0)	11.5 (4.2–13.7)	0.03
Maximum dosage (mcg/kg/min)	0.10 (0.07–0.16)	0.14 (0.09–0.25)	0.12 (0.10–0.18)	0.24
Duration of NE infusion (h)	15.0 (9.0–25.8)	6.2 (2.2–15.1)	17.0 (10.2 -24.0)	0.001
Total fluids (mL/h)	270 (166–537)	538 (182–1338)	251 (157–653)	0.01
Total fluids (mL/kg/h)	4.6 (2.8–8.8)	7.3 (2.9–19.5)	3.6 (2.8–9.2)	0.01
Time to antibiotic administration (h)	3.0 (1.5–6.0)	3.0 (1.8–5.0)	2.0 (1.1–3.5)	0.13

Values are presented as median (25th–75th interquartile range) or absolute (%). Group NE_CEI_, patients receiving a ceiling of care indication; Group NE_ICU_, patients admitted to ICU; NE, norepinephrine. ED, emergency department. All data reported are related to the ED stay (i.e., from ED admission to either ICU/ward discharge or death.

**Table 3:** Univariable and multivariable analysis on predetermined ED admission variables for the entire study population (*n* = 127).

	Univariable Analysis		Multivariable Analysis	
	OR (95% CI)	*p*-Value	OR (95% CI)	*p*-Value
Sex (Male)	1.26 (0.61–2.58)	0.536		
Age	0.97 (0.95–1.01)	0.206		
Body mass index	1.02 (0.95–1.10)	0.541		
SOFA score	0.89 (0.76–1.05)	0.163	0.85 (0.71–1.01)	0.058
Charlson	1.00 (0.78–1.29)	0.971		
MAP (ED admission	1.1 (0.99–1.03)	0.381		
Lactates (ED admission)	0.87 (0.72–1.06)	0.172		
Temperature	1.53 (1.17–2.02)	0.002		
Heart Rate	1.01 (0.99–1.02)	0.391		
Respiratory rate	1.03 (0.96–1.12)	0.402		
Glasgow coma scale	1.07 (0.85–1.34)	0.574		
Creatinine	0.81 (0.63–1.04)	0.105		
Total bilirubin	0.93 (0.81–1.08)	0.362		
WBC	0.96 (0.93–1.00)	0.046		
PLT	0.94 (0.70–1.28)	0.703		
PaO_2_	0.99 (0.95–1.02)	0.448		
PCO_2_	1.02 (0.97–1.09)	0.407		
HCO_3_	1.20 (0.95–1.51)	0.125		
Nora administration delay	1.00 (0.98–1.03)	0.869		
Fluids before ICU	1.03 (0.90–1.19)	0.643		
Antibiotics delay	1.10 (0.99–1.21)	0.075		
ICU admission	3.82 (1.35–10.81)	0.012	4.48 (1.52–13.22)	0.007

SOFA, Sequential Organ Failure Assessment; MAP, mean arterial pressure; ED, emergency department; WBC, white blood cells; PLT, platelets; PaO_2_, partial pressure of oxygen; PCO_2_, partial pressure of carbon dioxide; HCO_3_, bicarbonate; ICU, intensive care unit; OR (95% CI), odds ration with 95% confidence interval.

## Data Availability

The datasets used and/or analyzed during the current study are available from the corresponding author on reasonable request.
